# 1098. COVID-19 Reinfections in Older Adults: Risk Factors for Poor Outcomes

**DOI:** 10.1093/ofid/ofad500.071

**Published:** 2023-11-27

**Authors:** Elizabeth Lando-King, Keeley Morris, Scott Seys, Zachary Zirnhelt, Elle Talsma, Haley Wienkes, Kathryn Como-Sabetti, Stephanie Meyer

**Affiliations:** Minnesota Department of Health, St. Paul, MN; Minnesota Department of Health, St. Paul, MN; Minnesota Department of Health, St. Paul, MN; Minnesota Department of Health, St. Paul, MN; Minnesota Department of Health, St. Paul, MN; Minnesota Department of Health, St. Paul, MN; Minnesota Department of Health, St. Paul, MN; Minnesota Department of Health, St. Paul, MN

## Abstract

**Background:**

Older adults are at higher risk for hospitalization and death from COVID-19. The impact of COVID-19 reinfections on older adults is less clear. As more of the population experiences at least one COVID-19 infection, understanding risk factors associated with severe outcomes at reinfection is critical for improving patient care.

**Figure:**
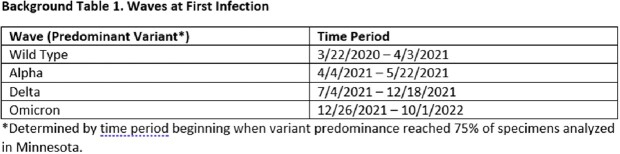

**Methods:**

We examined the risk of hospitalization, ICU admission, and death among MN residents aged 65+ experiencing 2^nd^ infections (reinfection) during the Omicron wave (12/26/2021-10/1/2022; n = 9311). We first performed log binomial regressions to examine risk of hospitalization, ICU admission, and death across the entire study period. We then stratified the sample by variant wave at 1^st^ infection (Table 1). All models were adjusted for time from 1^st^ infection to reinfection, gender (male vs. female), vaccination status at reinfection (vaccinated vs. unvaccinated), age group (65-74, 75-84, 85+), metro vs. outstate, and infection severity at 1^st^ infection (hospitalized vs. not hospitalized).

**Results:**

In the full model, people with 1^st^ infections during each of subsequent variant waves were at increased risk of hospitalization during reinfection relative to people whose 1^st^ infection occurred during the initial wild-type wave (Alpha: 1.27, 0.96-1.68, Delta: 1.29, 1.13-1.48, Omicron: 1.93, 1.72-2.18; Table 2). A similar effect was observed for ICU admission. In stratified analyses, age and severity at 1^st^ infection were significant predictors of hospitalization, ICU admission and death across some variant waves (Tables 3 and 4).

**Figure:**
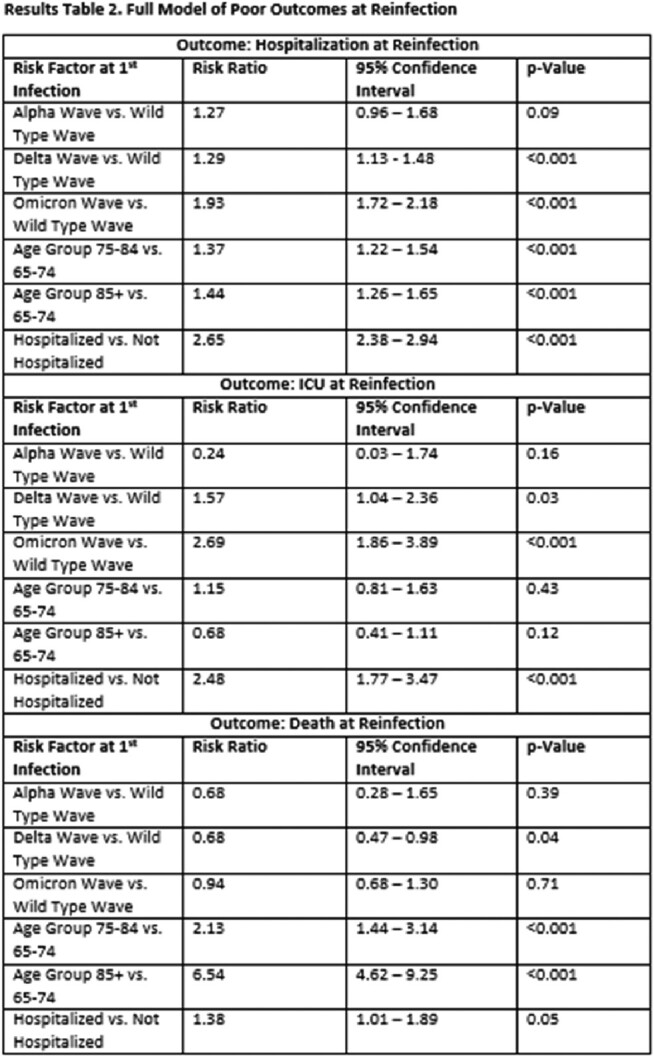

**Figure:**
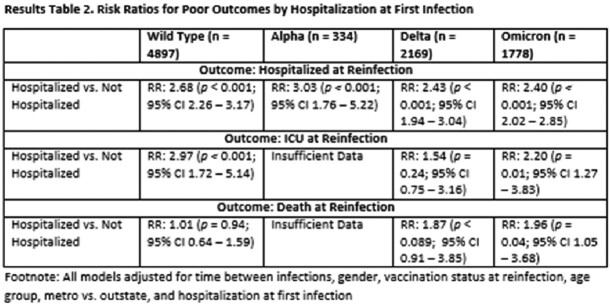

**Conclusion:**

In a preliminary examination of all Omicron reinfections, each subsequent variant wave of 1^st^ infection was associated with an increase in hospitalization. However, given the lack of substantial temporal overlap between variant waves, it is not possible to distinguish whether characteristics of the variant or time between 1^st^ and 2^nd^ infections is impacting magnitude of risk. Stratifying by variant wave allowed us to examine whether potential risk factors varied by variant of 1^st^ infection. Our stratified results indicate that greater age and hospitalization at 1^st^ infection significantly increase negative outcomes at reinfection regardless of the variant of 1^st^ infection.

**Figure:**
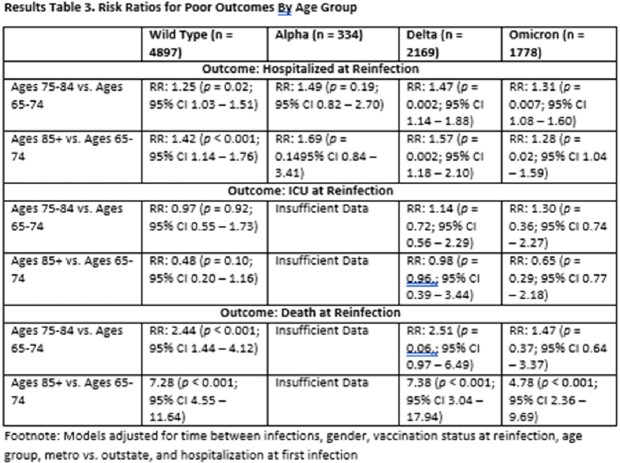

**Disclosures:**

**All Authors**: No reported disclosures

